# Software for Quantifying and Simulating Microsatellite Genotyping Error

**DOI:** 10.4137/bbi.s373

**Published:** 2009-11-24

**Authors:** Paul C.D. Johnson, Daniel T. Haydon

**Affiliations:** Division of Environmental and Evolutionary Biology, Institute of Biomedical and Life Sciences, University of Glasgow, Glasgow G12 8QQ, U.K

**Keywords:** microsatellites, genotyping error, allelic dropout, false alleles, maximum likelihood, software

## Abstract

Microsatellite genetic marker data are exploited in a variety of fields, including forensics, gene mapping, kinship inference and population genetics. In all of these fields, inference can be thwarted by failure to quantify and account for data errors, and kinship inference in particular can benefit from separating errors into two distinct classes: allelic dropout and false alleles. Pedant is MS Windows software for estimating locus-specific maximum likelihood rates of these two classes of error. Estimation is based on comparison of duplicate error-prone genotypes: neither reference genotypes nor pedigree data are required. Other functions include: plotting of error rate estimates and confidence intervals; simulations for performing power analysis and for testing the robustness of error rate estimates to violation of the underlying assumptions; and estimation of expected heterozygosity, which is a required input. The program, documentation and source code are available from http://www.stats.gla.ac.uk/~paulj/pedant.html.

## Introduction

Microsatellite genotype data underpin a wide diversity of genetic studies, in fields including individual identification in forensics, mapping disease genes, kinship inference and population genetics. All but the smallest microsatellite data sets will contain genotyping errors, which can be substantially misleading when undetected. Awareness of the implications of genotyping error has increased greatly in recent years, and there is an emerging consensus that error rates should be estimated and reported, particularly when performing error-sensitive analyses such as parentage analysis or individual identification (Bonin et al. 2004; Hoffman and Amos, 2005; Pompanon et al. 2005; DeWoody et al. 2006). Although the benefits of estimating and correcting for genotyping errors are most obvious when genotyping highly error-prone (e.g. non-invasive) samples, even low levels of genotyping error of 0.5–2% per genotype can mislead inference from microsatellite data (Feakes et al. 1999; Hoffman and Amos, 2005; Walters, 2005).

Ideally, error rates should be estimated by a combination of approaches, the most widely used being: (1) counting Mendelian inconsistencies in parent—offspring pairs; (2) comparing error-prone genotypes with high-quality reference samples; and (3) counting mismatches between duplicated genotypes. The first two approaches will generally yield more reliable estimates than the third, but may not be feasible because pedigree data and reference samples will often be unavailable. The third option of re-genotyping a random subset of samples at all loci is widely recommended (Bonin et al. 2004; Hoffman and Amos, 2005; Pompanon et al. 2005; DeWoody et al. 2006) and will almost always be feasible but suffers from two disadvantages. Firstly, when errors are frequent a non-negligible proportion of repeat genotypes will sustain errors in both genotypes, leading to undercounting of errors (e.g. due to an underlying heterozygous genotype of *AB* being genotyped twice as *AA*). Secondly, many analyses benefit from breaking down single error rates into two distinct classes of error—allelic dropout and false alleles (Broquet and Petit, 2004)—which is not straightforward when both duplicates are error-prone.

Pedant is a program for MS Windows that implements a maximum likelihood method for estimating rates of allelic dropout and false allele error from microsatellite genotype data (Johnson and Haydon, 2007). Allelic dropout occurs when a heterozygote is genotyped as a homozygote due to the random failure of one allele to PCR-amplify, and a false allele occurs when an allele is mis-genotyped as any other allele (Broquet and Petit, 200). Allelic dropout and false allele error rates can be estimated as per-genotype probabilities, but here we treat them as per-allele probabilities. In a single genotype, ɛ_1_ is the probability of an allele dropping out, and ɛ_2_ is its probability of being mis-typed as another allele (Wang, 2004). Conversion between the per-allele and per-genotype rates is simple, and both are calculated by Pedant.

Division of errors into allelic dropouts and false alleles is useful not only because allelic dropouts are typically more frequent than false alleles (Broquet and Petit, 2004), but also because the two classes of error affect analyses in fundamentally different ways (Wang, 2004; Hadfield et al. 2006). For example, consider a simple paternity analysis using one locus at which every allele has a 7% probability of error. There are three individuals: one offspring (genotype *AB*), and only two possible fathers (genotypes *CD* and *CC*). Neither candidate father matches the offspring, so an error must be invoked, but because we cannot say in which candidate the error is more likely to have occurred, the data are uninformative. However, suppose we can break down the total error probability into ɛ_1_ = 5% for allelic dropout and ɛ_2_ = 2% for false alleles. We will then have greater confidence in genotypes *AB* and *CD* than in *CC*, because only *CC* can have been affected by allelic dropout (assuming only one error per genotype), and attributing *AB* or *CD* to error would require us to invoke a less likely class of error. Thus, when separate error rates are known, the data become more informative: the candidate with genotype *CC* is the more likely father. This principle has been successfully applied to pedigree reconstruction (Wang, 2004; Hadfield et al. 2006) and individual identification (Kalinowski et al. 2006).

Unlike estimating a single error rate, estimating ɛ_1_ and ɛ_2_ is not a simple exercise in counting errors because when both repeat genotypes are error prone the class of error responsible for a mismatch can be ambiguous. For example, the mismatch *AA*/*AB* could be caused by allelic dropout in an underlying genotype of *AB* (giving *AA*) or a false allele in an underlying *AA* (giving *AB*). Instead, Pedant counts different types of matching and mismatching duplicate genotypes (*AA*/*AA*, *AA*/*AB*, *AB*/*AB*, *AB*/*AC*, etc) and searches for the pair of error rates that maximises the likelihood of the observed counts (Johnson and Haydon, 2007). For example, a duplicated data set with few *AA*/*AB*-type mismatches and many of type *AB*/*AC* supports a low allelic dropout rate and a high false allele rate.

The first step in using Pedant is to ascertain whether the data fit the underlying assumptions of the error model. The principal assumption is that the probability that an underlying genotype is heterozygous (observed heterozygosity, *H_o_*) is known. However, as *H_o_* will be biased by allelic dropouts and false alleles, Pedant uses instead expected heterozygosity, *H_e_*, which will equal *H_o_* only when the study population is in Hardy Weinberg equilibrium (HWE). Deviations from HWE, such as a substantial heterozygote deficit (*F_IS_* > 0.1), that cannot be corrected for using prior knowledge will lead to biased estimates (Johnson and Haydon, 2007). Known or suspected population structure can easily be corrected for by using the average within-subpopulation *H_e_* in place of the global *H_e_*. Pedant also assumes that errors occur with equal probability across samples and that each allele of a heterozygote is equally likely to drop out. Pedant is fairly robust to deviations from both of these assumptions, but a combination of severe variation in sample quality (Pompanon *et al.* 2005), short allele dominance (Wattier *et al.* 1998) and high allelic dropout rate (ɛ_1_ > 0.1) can lead to underestimation of both ɛ_1_ and ɛ_2_. Nevertheless, testing of Pedant on real and simulated data suggests that the method is robust to modest deviations from its underlying assumptions (Johnson and Haydon, 2007).

The second step in using Pedant is to decide how many genotypes to duplicate at each locus. This will depend mainly on the degree of accuracy required by the downstream analysis, but approximate estimates will generally be adequate (SanCristobal and Chevalet, 1997; Sieberts et al. 2002; Wang, 2004). The most important factor in determining accuracy is generally the number of visible errors in the input data. Thus, error rate estimation will be most accurate from data with high error rates and high *H_e_* (because at low *H_e_* many dropouts will be hidden), and when these conditions do not apply sample size should be increased to compensate. If average cross-locus error rate estimates are sufficient, all loci can be pooled as a single locus, so with 10 loci perhaps only 25 to 50 duplicate genotypes per locus might be necessary. On the other hand, if locus-specific estimates are required then at least 100 repeats per locus might be required. These figures are very rough guidelines only. Specific scenarios can be explored using Pedant’s simulation function, which allows users to simulate the estimation of error rates from multiple data sets while varying parameters (ɛ_1_, ɛ_2_, *H_e_*, *F_IS_* and sample size). For each error class the individual estimates and their mean and standard deviation are plotted, allowing rapid visual assessment of estimation accuracy under a variety of parameter combinations. Five hundred simulated data sets can be analysed in about 30 seconds.

When the data have been deemed suitable for analysis using Pedant and sample size has been decided, samples should be selected at random and repeat-genotyped blind at all loci. Pedant accepts genotypes in tab-delimited text format, which is easy to create from a spreadsheet. Alternatively, a Pedant input file can be converted from genepop format using an R script included in the download. The input file must contain *H_e_* estimates for each locus. To minimise the effect of sampling error, *H_e_* should be estimated from the whole data set, not just the subset that has been duplicated. *H_e_* (and its standard error) can be calculated in Pedant.

**Figure 1.**
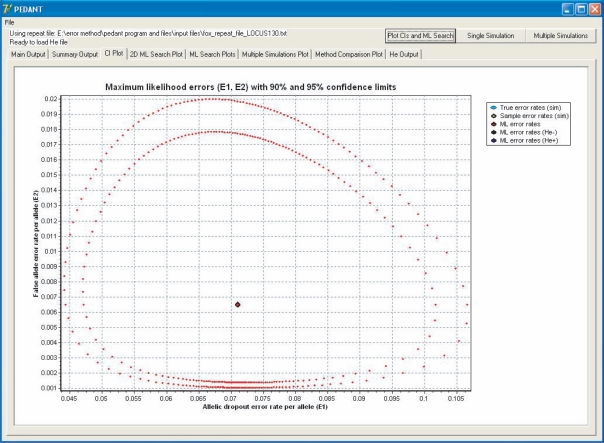
The Pedant interface, showing ML error rate estimates for allelic dropout and false alleles with 90% and 95% confidence regions.

When the input file has been loaded, Pedant locates the maximum likelihood (ML) estimates, of ɛ1 and ɛ2, which takes about one second per locus, and outputs the estimates together with 95% confidence intervals calculated using the relative log-likelihood function. Estimates and confidence regions can also be plotted for individual loci (Fig. 1). Details of the ML search algorithm are given in the program documentation.

An alternative method for estimating ɛ_1_ and ɛ_2_ across markers is available in MasterBayes, an R package for Bayesian pedigree reconstruction (Hadfield et al. 2006). MasterBayes samples the posterior distributions of ɛ_1_ and ɛ_2_, from which point estimates and credible intervals can be obtained using summary statistics describing the relevant marginal distribution. Although Pedant and MasterBayes differ in their approaches to parameter estimation (maximum likelihood versus Bayesian), their underlying error models are similar and they would be expected to reach similar estimates. We compared the two programs using two simulated data sets: low error (ɛ_1_ = 0.01, ɛ_2_ = 0.005) and high error (ɛ_1_ = 0.05, ɛ_2_ = 0.02). The low rates are typical of highly automated genotyping while the higher rates represent smaller, more error-prone studies (Pompanon et al. 2005). We set ɛ_1_ higher than ɛ_2_ because allelic dropouts are generally more frequent than false alleles (e.g. Broquet and Petit 2004). Comparison of the mean error estimates across 100 loci (Table 1) shows that both programs reach broadly similar and accurate estimates, particularly at higher error rates (for the reasons noted above). For both programs bias was highest in estimating the lowest of the four error rates (ɛ_2_ = 0.005), with MasterBayes overestimating (ɛ̂_2_ = 0.0066, bias = 31%) and Pedant underestimating (ɛ_2_ = 0.005, ɛ̂_2_ = 0.0041, bias = −18%). Overall Pedant recovered the population error rates more accurately, although it should be noted that the accuracy of both programs will vary with other parameters such as *H_e_* and sample size. For the small sample sizes that are likely to be feasible, by far the greatest source of estimation error will be sampling error (as reflected by the width of the interquartile range), which is beyond the control of either method. Given that the two programs produce similar results, the choice of which to use should be guided by more pragmatic considerations. Pedant is simpler to use, in that it has a user-friendly interface (Fig. 1) and does not require familiarity with computer programming. Moreover, production of locus-specific estimates, which are used by some analyses (e.g. Wang, 2004), is more straightforward. However, error rate estimation is more flexible in MasterBayes because it can use more than two repeat genotypes and it allows the incorporation of pedigree information. In addition, like other R programs it has the useful property of being adaptable by users with some knowledge of the R programming language. Thus, the choice of program will depend upon the experience and requirements of the user.

The MS Windows executable program, documentation, input file converter and source code for Pedant (written in Borland Delphi version 7.0) are available from http://www.stats.gla.ac.uk/~paulj/pedant.html.

## Figures and Tables

**Table 1.** Summary statistics assessing the performance of the programs MasterBayes and Pedant in estimating error rates from two simulated data sets.

**Simulated data set**^a^	**Program**^b^	**Error rate parameter**	**Mean estimate (95% CI of mean)**	**Median estimate (interquartile range)**	**Bias**
Low error rates (*n* = 100 loci)	MasterBayes	ɛ_1_ = 0.01	0.0118 (0.0105, 0.0130)	0.0111 (0.0072, 0.0160)	17.9%
		ɛ_2_ = 0.005	0.0066 (0.0058, 0.0073)	0.0056 (0.0038, 0.0085)	31.3%
	Pedant	ɛ_1_ = 0.01	0.0107 (0.0095, 0.0119)	0.0098 (0.0061, 0.0147)	7.0%
		ɛ_2_ = 0.005	0.0041 (0.0034, 0.0048)	0.0032 (0.000, 0.0062)	−18.3%
High error rates (*n* = 100 loci)	MasterBayes	ɛ_1_ = 0.05	0.0526 (0.0496, 0.0556)	0.0517 (0.0411, 0.0619)	5.2%
		ɛ_2_ = 0.02	0.0228 (0.0211, 0.0244)	0.0221 (0.0169, 0.0287)	13.8%
	Pedant	ɛ_1_ = 0.05	0.0511 (0.0483, 0.0539)	0.0516 (0.0409, 0.0598)	2.2%
		ɛ_2_ = 0.02	0.0193 (0.0177, 0.0208)	0.0178 (0.0140, 0.0248)	−3.7%

a For each data set 100 duplicate genotypes were simulated in MasterBayes from 100 loci with 10 alleles per locus. Allele frequencies for each locus were generated randomly from the broken stick distribution.

b In MasterBayes locus-specific error rates were estimated as the median of the relevant marginal distribution based on 100,000 MCMC iterations (after discarding 10,000 burn-in iterations). In Pedant the ML estimates located during 10,000 search iterations were used.
